# The Involvement of Serotonin in the Obesity Pathway—A Last Decade Systematic Review of the Literature

**DOI:** 10.3390/ijms26073081

**Published:** 2025-03-27

**Authors:** Radu-Cristian Cîmpeanu, Emilia-Mariana Caragea, Lorena-Maria Mustață, Dragoș Forțofoiu, Ioana-Gabriela Dragne, Raluca-Elena Alexa, Anastasia Balta, Alexandr Ceasovschih, Laurențiu Șorodoc, Larisa-Daniela Săndulescu

**Affiliations:** 1 Doctoral School of University of Medicine and Pharmacy of Craiova, 200349 Craiova, Romania; 2 Doctoral School of “Grigore T. Popa” University of Medicine and Pharmacy, 700115 Iasi, Romania; 3 Faculty of Medicine, “Grigore T. Popa” University of Medicine and Pharmacy, 700115 Iasi, Romania; 4 Faculty of Medicine, University of Medicine and Pharmacy of Craiova, 200349 Craiova, Romania

**Keywords:** serotonin, 5-hydroxytryptamine, obesity, weight gain

## Abstract

Obesity represents a complex, multifactorial syndrome that represents a high burden for public health systems worldwide. Serotonin is an important factor in feeding behavior and weight regulation and their interplay implies multiple mechanisms that could explain the correlation with obesity, so understanding these interconnections is essential for developing targeted therapeutic strategies. A systematic review of the literature was conducted using PubMed and Scopus databases, using articles published between 1 January 2015 and 1 December 2024, based on predefined inclusion and exclusion criteria. After the selection process, 22 studies were selected for detailed analysis, focusing on the role of serotonin in obesity. Serotonin significantly influences appetite control and energy homeostasis through multiples pathways, including insulin resistance, high-fat diets, gut microbiota, low-grade inflammation, interferences with tryptophan metabolism, psychiatric modifications, genetic alterations of serotonin receptors, serotonin implications in eating behavior, and neurohormonal regulation of appetite. This review highlights the multidimensional characteristics of the serotonin–obesity association, along with its significance in metabolic and psychiatric pathologies. In order to develop more efficient methods for managing obesity, future studies should concentrate on serotonergic regulation and complex management strategies involving the neurohormonal axis.

## 1. Introduction

Obesity is a complex, heterogeneous, chronic, latent, and progressive disease, which causes multiple organ damages, especially regarding the liver, the heart, kidneys, and the pancreas, alongside with respiratory dysfunction [1,2,3,4]. Moreover, it is already known that obesity is a worldwide problem, an “epidemic disease” as declared by The World Health Organization. Now, several directions about obesity are studied, including pathophysiology pathways, in order to obtain complex and suitable therapeutic targets for more efficient management.

Although improvements in the diagnosis and treatment of patients with obesity have been recorded, its incidence trend continues to increase. Obesity represents a cumulative disease that appears through genetic factors, and interaction between modern lifestyle and chemical exposure [5,6,7].

One of the recently researched mechanistic pathways implies serotonin involvement in obesity. In the last decade, this correlation has added to the existing knowledge about obesity, including eating behavior influences, such as the activation of the anorexigenic α-melanocyte stimulating hormone, as well as orexigenic neuropeptide Y (NPY) and Agouti-related peptide inhibition, centered by tryptophan (TRP) metabolism [8,9,10].

Serotonin (5-hydroxytryptamine, 5-HT) is a biogenic amine, known as a neurotransmitter and recognized for its multiple functions in gastrointestinal motility, vascular tonus maintenance, and platelet function, and for its role in metabolic homeostasis.

Serotonin biosynthesis from TRP occurs in enterochromaffin cells of the digestive tract and neurons in the central nervous system [11]. Functionally, serotonin has a major localization of 90–95% in the gastrointestinal tract of the human body. It is stored in granules at the basal and apical ends of the cells [11,12]. In platelets, serotonin is a signal molecule that is involved in the response to damage endothelium and ischemia [10].

The smaller part of serotonin (less than 1%) is secreted in the central nervous system, where it is released and implicated in synapse functions, as well as in emotional responses, including sleep, temperature regulation, cognition, anxiety, and appetite [10,13,14,15].

Regarding serotonin receptors, fourteen distinct 5-HT receptors have been described [11]. In terms of the insulin secretion and glucose metabolism implication, 5-HT1ARs, 5-HT1DRs, 5-HT1FRs, 5-HT2BRs, 5-HT3Rs, and 5-HT5ARs are present at this level, which act as an autocrine signal modulating β-cells’ function and proliferation [16,17,18,19,20,21].

Normal serotonin secretion is important for proper appetite and body-weight regulation, and increased secretion is responsible for weight gain and dysregulation processes that lead to obesity development [16,22,23].

So, we can conclude that serotonin is involved in regulating energy homeostasis through its involvement in feeding behavior, which could be targeted as an optimal therapeutic option in obesity management [24,25,26,27,28]. The worldwide impact of obesity and the necessity of establishing optimal strategies are the reasons that prompted us to summarize this pathway, aiming to contribute to centralized information that could help in developing new therapeutic strategies.

## 2. Methods

We conducted a systematic review of the literature regarding the involvement of serotonin in the obesity mechanism, reporting relevant items about this topic. We registered the review protocol under the CRD42025650050 number on Prospero, and, furthermore, using the Population, Intervention, Comparison, Outcome, and Study Design, we developed the strategy that guided our study rationale.

### 2.1. Research Question and Search Strategy

We conducted a qualitative systematic search of the literature, based on the PubMed and Scopus databases, using the “serotonin AND obesity” search criteria. We used Boolean operators such as “AND” in the search process, targeting free full articles written in English and studies carried out on adults, and we excluded preprints. Also, we limited our search to keywords, titles, and abstracts, and without specific searching due to the existence of exclusion criteria.

We obtained 68 articles from PubMed and 159 articles from Scopus.

### 2.2. Inclusion Criteria

The inclusion criteria were original full-text articles, namely randomized controlled trials and clinical trials that were published in English from 1 January 2015 to 1 December 2024 conducted on human populations that reported information about the role of serotonin in the pathogenesis of obesity.

### 2.3. Exclusion Criteria

The exclusion criteria were articles that were case reports, reviews, meta-analyses, letters to the editors, or duplicates, that lacked originality, that were published in languages other than English, that were conducted on non-human populations or only on cell cultures or cell lines, or evaluated the diagnosis and therapeutic processes; we also excluded child, adolescent, psychology, mental disease, and drug influences.

### 2.4. Study Selection

Studies that met the eligibility criteria (1) included human patients with obesity and serotonin assessment; (2) evaluated the relationship between serotonin involvement and obesity; and (3) provided sufficient information such as 95% confidence intervals or, at least, *p*-values. Studies were excluded if they (1) were redundant publications; (2) provided insufficient or incomplete data; or (3) were case reports, letters to the editor, meeting abstracts, expert opinions, or reviews.

**Figure 1:**
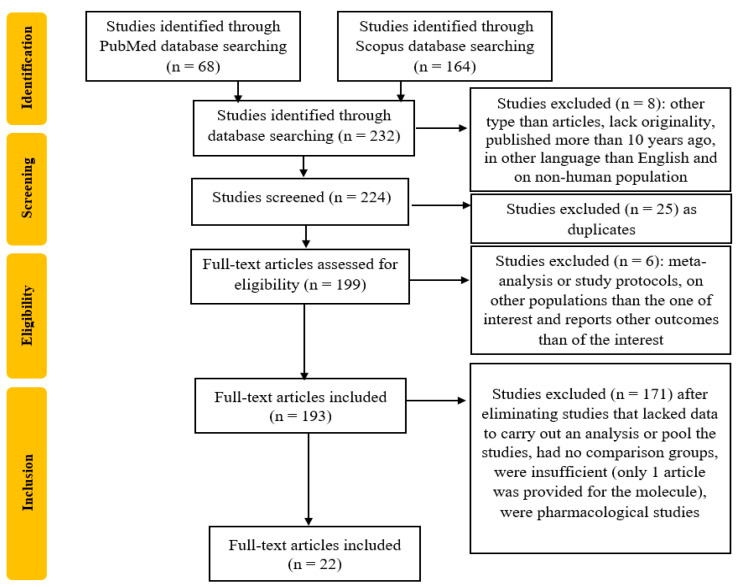
Flowchart of the study selection process.

The study selection process is detailed in Figure 1. From the 232 studies initially selected, 8 were excluded due to lack of originality, having a publication date more than 10 years ago, being conducted on non-human study populations, or being written in a language other than English. Next, 25 duplicate studies and 6 meta-analyses, study protocols, or reports on unrelated outcomes were removed. From the remaining 193 studies, 171 were excluded due to insufficient data or being pharmacological studies.

### 2.5. Data Extractions

Two researchers extracted the studies’ titles and abstracts, screened them for relevance to the present study theme, searched for the presence of at least one analysis of a human population with obesity, and selected the relevant ones by performing cross-screening. This process was performed using a standardized form, which included information about publication year, study type, study aim, inclusion and exclusion criteria, outcomes, and conclusions. If any disagreements occurred in the selection process, these were settled by a third reviewer.

We also performed a manual search of the databases to identify other potentially useful articles missed by our search strategy and identified an article.

### 2.6. Risk of Bias

Assessment bias assessment was carried out using the Newcastle-Ottawa scale (NOS) for cross-sectional studies and Risk of Bias 2 scale (RoB2) for randomized controlled studies (RCT). NOS and RoB2 were conducted by three reviewers. They independently assessed the quality of the chosen studies using a star rating system that evaluated the selection, compatibility, and outcome criteria for articles. The results are shown in Table 1 and Table 2.

From the twenty-two selected articles, we collected data regarding their specific information that was presented, which is centralized in Table 3.

### 2.7. Strategy of Data Synthesis

After the selection process and the articles’ evaluation by the reviewers, 22 articles were included. The process is summarized in Figure 1.

Furthermore, a narrative synthesis of the findings from the studies centered on serotonin’s involvement in human populations with obesity and its relationship with the disease was realized. All the studies included in this systematic review were screened for statistical significance according to serotonin’s implication in obesity; for that matter, all the correlations between serotonin and anthropometric measurements, diet habits, metabolic factors, inflammatory biomarkers, gut microbiota, genetic polymorphisms, and neurotransmitters that had statistical significance were extracted, and are synthesized in Table 4. Because the studies were expected to be heterogeneous in terms of study design, quality and screening methods, interventions, and outcomes described, a narrative synthesis was performed using text and tables in order to provide a descriptive summary and explanation of the study characteristics and findings. The current article refers to approximately the last 10 years of developments in understanding serotonin’s involvement in the pathogenic mechanisms in human populations with obesity, reviewing recent pathophysiology mechanisms that have been established and described on this topic.

## 3. Results and Discussion

In the last decades, researchers have investigated the role of serotonin in metabolic pathways, trying to find its reciprocation in weight regulation disturbances. As can be observed, there are many studies, some of them being conclusive about the influence of this neurotransmitter and its correlation in obesity, through lifestyle, genetic profiles, metabolic features, and other neurotransmitters that influence the normal way of weight maintenance.

### 3.1. Correlation Between Metabolic Factors, Serotonin and Obesity

TRP is an essential amino acid that cannot be synthesized by the human body, and is involved in protein synthesis and inflammation. Its metabolites are associated with indoleamine 2,3-dioxygenase (IDO-1) activity, which is increased by serotonin reduction.

Moreover, TRP is oxidized via kynurenine (KYN) and indole pathways, leading to metabolites that cross the blood–brain barrier and interfere with emotions, cognition, and appetite, among other neurological processes.

Increased pro-inflammatory cytokine levels brought on by inflammatory diseases like obesity can activate IDO-1, an enzyme that catalyzes the conversion of TRP to KYN. Serotonin availability is reduced as a result of this activation. According to studies, people who are obese have higher KYN/TRP ratios, which suggests that they have less serotonin production and more IDO-1 activity. Peripheral serotonin does not cross the blood–brain barrier, but can interfere with central serotonin production via vagal signaling.

Depressive symptoms have been linked to elevated levels of kynurenine and its metabolites, which further affect eating habits and lead to obesity. This cycle produces a feedback loop in which inflammation is made worse by obesity, which further diminishes serotonin and increases food intake.

Insulin resistance induced by high-fat diets involves multiple mechanisms, such as phosphorylation of insulin receptor substrate 1, a process determined by branched-chain amino acids (BCAAs) through the rapamycin (also known as the “mTOR pathway”—Mammalian Targets of Rapamycin) and S6-kinase 1 pathways, accumulation of oxidized BCAA metabolites and mitochondrial dysfunctions, or the accumulation of xanthurenic acid and kynurenic acid, metabolites of the indole pathway resulting from adipose tissue catabolism of tryptophan.

Obese individuals have markedly changed aromatic and BCAA pathways, which may have implications for serotonin activity and levels. Dysregulation of these pathways could make it more difficult for the body to regulate hunger and energy expenditure, which may lead to the development of obesity.

Patients with obesity have an increased production of proinflammatory cytokines, with both obesity and high-fat diets maintaining a low-grade inflammatory state.

Moreover, serotonin is involved in β-cell mass expansion during puberty through the growth hormone, its receptor, and insulin-like growth factor-1, which are associated with insulin resistance, increasing the risk of late-onset metabolic syndrome later in life.

As is well known, lower serotonin levels are linked to higher rates of anxiety and depression, but this effect might cause overeating as a coping strategy. However, this can lead to a vicious cycle whereby eating more food further lowers serotonin levels, worsening psychological distress and obesity.

There are several hormones involved in appetite regulation, such as leptin, ghrelin, peptide YY, glucagon-like peptide 1, insulin, and glucagon. Their release is modulated by food composition, fasted eating, and low-grade inflammation, and they modulate the serotonergic and dopaminergic systems.

### 3.2. Correlation Between Gut Microbiota, Serotonin, and Obesity

Increased levels of *Prevotella* spp. in the microbiome lead to dysregulations and insulin resistance through BCAA formation. This specific gut microbiota population in obese patients, rich in *Prevotella* spp. and *Bacteroides* spp., influences the conversion of TRP into serotonin, thereby diminishing serotonin levels, leading to increased appetite signaling and weight gain.

Dietary factors, particularly increased protein intake, lead to high levels of TRP in plasma, suggesting that dysbiosis induced by dietary habits interfere with serotonin levels through the gut microbiota.

Fiber-rich diets can increase the synthesis of short-chain fatty acids (SCFAs), such as butyrate, which has been demonstrated to improve insulin sensitivity and serotonin signaling. This implies that dietary changes targeted at enhancing gut health may positively impact serotonin levels, which in turn may influence hunger control and weight management.

Additionally, the location and function of enterochromaffin cells (ECs) may be changed in obese people, which can result in dysregulated hormone release and difficulties in controlling eating. Furthermore, the human microbiota promotes serotonin biosynthesis from ECs [52]. Thus, weight gain can result from EC dysfunction, which can increase hunger and food intake.

### 3.3. Correlation Between Genetics, Serotonin, and Obesity

Numerous eating disorders, which are frequently linked to obesity, have been associated with variations in the serotonin transporter gene, such as SLC6A4, which codes for the serotonin transporter (SERT) and interferes with serotonin reuptake in the brain. It has been demonstrated that SERT availability in the prefrontal cortex is associated with reward and decision-making, which interferes with food intake.

Genetic polymorphisms that impact serotonin transmission, such as rs6265 for brain-derived neurotrophic factor (BDNF) or SLC6A4 for 5-HTTLPR, may contribute to the development of obesity by increasing food intake, mood disturbances, and cognitive impairment.

Reduced SERT expression has been associated with the presence of the 5-HTTLPR short allele. This could result in elevated synaptic serotonin levels and altered reward processing in relation to food intake. Moreover, serotonin signaling can also be significantly influenced by epigenetic changes, such as DNA methylation of 5-HTTLPR. Higher 5-HTTLPR methylation rates have been linked to lower SERT availability in the prefrontal cortex, a part of the brain involved in eating behavior decision-making and impulse control.

All these alterations in epigenetics suggest that serotonin signaling plays an important role in reward-related behaviors, influencing weight gain.

### 3.4. Correlation Between Lifestyle, Serotonin and Obesity

From the total of 22 included articles, 6 presented traits about lifestyle. They revealed the intricate and multifaceted connection between obesity, serotonin levels, and weight regulation through lifestyle influences. For example, in a cohort study published in 2023, elevated body weight and obesity-related parameters were strongly associated with reduced serotonin levels, which in turn correlated with poorer sleep quality. After one month of initiated treatment, the levels of serotonin moderately increased. Over a six-month period, significant improvements were observed in Body Mass Index (BMI), mental health, sleep patterns, and metabolic markers, particularly in groups showing notable increases in serotonin levels. However, a negative correlation was observed between serotonin and obesity, suggesting that while serotonin plays a role in weight regulation, no direct correlation with obesity could be ascertained. In another study conducted on 37 patients, which also profiled the hormonal background, it was revealed that low serotonin levels linked to obesity might result in compensatory behaviors like overeating to raise serotonin and dopamine levels. Patients exhibited significant psychosocial and behavioral issues, such as depression and anxiety, linked to low serotonin levels and lifestyle factors.

Moreover, in a cross-sectional study that included 46 nurses that work in a night-shift program, it was once again highlighted that for this employee category, the BMI was higher and the serotonin level was lower.

That suggests that serotonin secretion is influenced by many other factors like psychosocial stressors and disruptions to circadian rhythms.

Thus, a reciprocal relationship was observed between BMI and serotonin. In order to treat obesity, patients received patient-centered motivational counseling about maintaining a healthy diet and lifestyle, exercising, maintaining good sleep hygiene, and adjusting risk factors in compliance with current clinical guidelines. Dietary adjustments were recommended to achieve efficient results in improving their health.

### 3.5. Correlation Between Neurotransmitters, Serotonin, and Obesity

Changes in BMI are closely linked to alterations in the structure and function of the brain, especially in the reward and cognitive control systems. Dopamine and serotonin imbalances affect signaling pathways and enhance craving and food intake, particularly of high-calorie foods. According to neuroimaging studies, patients with obesity exhibit abnormalities in areas like the cingulate cortex and frontal lobe, which are involved in cognitive control and eating-related decision-making processes. Reduced SERT availability in the diencephalon lead to glucose metabolism impairments [53]. 

SERT levels are frequently lower in obese people, which may result in less serotonin being available in the central nervous system. The brain’s capacity to properly control food intake may be compromised by this decrease in SERT, which could result in overeating and obesity. Furthermore, certain receptors, such as the 5-HT1B and 5-HT2C receptors or the 5-HT6 receptor, mediate the anorexic effects of serotonin.

Fasting typically increases SERT availability, but in obese individuals, this effect is diminished, indicating a long-term disruption in serotonergic transmission that could be a factor in their ongoing overeating and weight gain. Serotonergic signaling plays a key role in the striatum, a crucial region involved in reward processing. Decreased serotonergic activity may promote overeating by intensifying cravings for high-calorie foods. Increased serotonergic transmission, on the other hand, has been linked to reduced food intake and improved satiety, suggesting that serotonin is involved in both reward-driven and homeostatic eating behaviors.

Higher levels of glycosylated hemoglobin (HbA1c), a sign of persistently elevated blood glucose, are inversely correlated with serotonin transporters availability in the hippocampus, a brain region responsible for hunger regulation. Given that lower levels of BDNF have been associated with both an increased risk of obesity and decreased cognitive performance, the association between SERT availability and BDNF implies that BDNF may be involved in influencing serotonin signaling and energy metabolism. Impaired hippocampal function due to serotonin dysregulation can result in learning and memory deficits related to satiety and food cues.

Enteroendocrine cells (EECs), part of the nervous system of the gastrointestinal tract, release different hormones and neurotransmitter, in response to luminal content. Peripheral serotonin, released by enterochromaffin cells, activates the gastrointestinal tract, leading to motility and secretion of molecules that activate the vagal system and induce the increase in central serotonin and loss of appetite, this leading to weight loss.

### 3.6. Serotonin Pathways in Obesity Development

**Figure 2:**
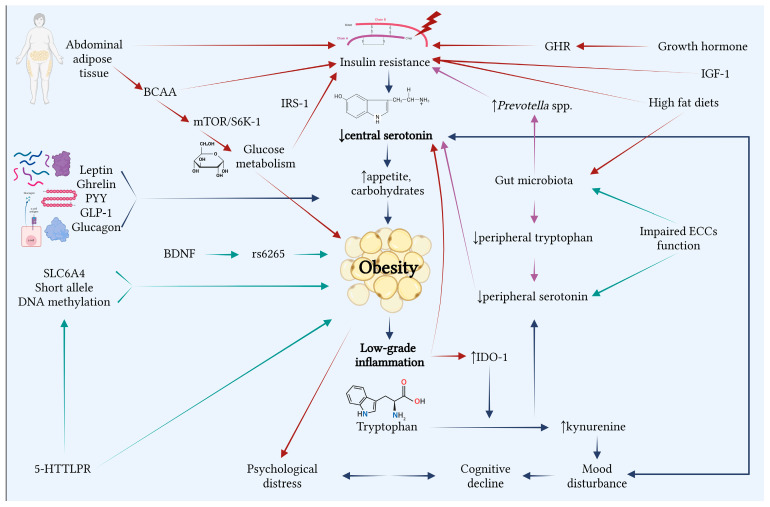
Interrelations between serotonin and obesity (Created with BioRenders.com). Numerous factors, including genetics, neurotransmitters, gut microbiota, metabolism, and psychological disorders, connect obesity to serotonin. The primary contributor is low central serotonin levels, which increase appetite, particularly for carbohydrates, and contribute to and perpetuate obesity by causing low-grade inflammation. Obesity and psychological distress have a reciprocal relationship that results in mood disorders and cognitive deterioration. The gut microbiome is crucial because low tryptophan levels cause peripheral serotonin levels to drop, which maintain the low concentrations of central serotonin, the vagal signaling responsible for the central secretion being diminished. Increased hunger and low-grade inflammation driven by obesity and insulin resistance, as well as the interference of abdominal adipose tissue with many processes that sustain obesity, are the mechanisms by which hormones and serotonin are linked. Obesity is also caused by mutations in the genome, the most significant of which are 5-HTTLPR genetic variations. The various facets of obesity are shown by the connections between all of these linkages.

Serotonin levels are influenced by various factors, with studies supporting its influence in mood regulation, metabolic processes, and appetite control. Recent research highlights 5-HT’s role in obesity development, where it mediates inflammatory pathways, food intake and energy expenditure (Figure 2).

The primary mechanism involved is chronic inflammation, which is induced by obesity itself but also driven by psychological distress [30], diet type [30], gut microbiota composition [35], and alterations in the brain network activity [35].

Studies have shown that serum 5-HT levels are lower in patients with obesity compared with lean individuals [28,39]. Moreover, the work schedule, especially night shifts, may influence 5-HT levels [31]. Interestingly, higher 5-HT levels were observed in male patients with hypertriglyceridemia [36], following weight loss [30] or after 6 months of patient-centered motivational approach [33].

TRP’s metabolism via KYN pathway, assessed by increased KYN levels and an elevated TRP/KYN ratio (as a surrogate for IDO-1 pathway’s activation), has been positively correlated with BMI [39,46]. Additionally, decreased fecal TRP levels, alongside increased *Prevotella*/*Bacteroides* ratio were associated with higher BMI [35].

The interplay between lipid and glucose metabolisms affects the SERT availability. However, findings on the relationship between SERT availability and BMI are contradictory [37,40,48], but one possible connection between glucose metabolism and SERT function could be BDNF [41]. Furthermore, lower SERT binding was associated with insulin resistance [48] and increased levels of HbA1c [41], suggesting that glucose dysregulation may contribute to cognitive impairment.

Although gut microbiota alteration appears to impact DAT binding rather than SERT binding, the results from studies show that patients with obesity exhibited alteration in brain connectivity, especially in reward-related regions [29], with a negative correlation between BMI and SERT availability [45]. Also, patients with obesity showed no response to fasting compared to lean individuals regarding hypothalamic SERT levels [40].

Furthermore, the metabolite profile shows certain BCAAs and AAAs involved in obesity mechanisms, correlating with VAT’s implication in metabolic pathways in obesity [36].

5-HT plays an important role in obesity through its influence on energy homeostasis and chronic inflammation. The included studies suggest 5-HT’s implication in obesity development, but future research is required to assess the pathways involved and to develop targeted therapeutic approaches to manage weight and obesity-related risk factors.

### 3.7. Limits

The current article is a systematic review of research conducted during the last decade regarding the involvement of 5-HT in obesity development. Based on the selected studies and their analysis, our study has the following limitations: (a) the majority of the studies are cross-sectional, observational studies, which demonstrated the correlation between 5-HT and BMI, along with metabolic dysregulation and brain activity changes; (b) all the studies had different inclusion and exclusion criteria, and the results cannot be generalized in the population; (c) most of the studies had a small sample size; (d) no follow-up of the patients was conducted; and (e) self-reported food diaries, sleep, and mental health data could be a source of bias.

### 3.8. Research Future Directions and Perspective Targeted Treatments

From health systems’ perspectives, it is essential to track shifts in the nutritional status of individuals and explore the possible causes and consequences of these changes. Obesity eradication strategies include lifestyle modifications, pharmacological interventions, and metabolic surgeries [54].

In the human body, the hypothalamus regulates body weight by combining neuronal inputs to regulate the appetite using signals like leptin and insulin [53,55]. It is known that leptin concentrations increase proportionally with fat tissue [56]. Reduced levels of insulin and leptin are accompanied by increased concentrations of glucagon, growth hormone, and catecholamines [57]. Functionally, leptin inhibits diencephalic nitric oxide synthase activity and increases brain serotonin metabolism [58]. The complex interactions between the brain, gut, pancreatic islets, and adipose tissue in regulating appetite and the reward center may alter the insulin/serotonin (5-HT)/leptin axis in obesity, potentially impacting the density, function, or affinity of SERT in distinct ways [59,60].

Another recently described mechanism for obesity is represented by gut microbiota intervention. Studies have demonstrated that typical ratios between specific human gastrointestinal microbiota, like the *Prevotella*/*Bacteroides* ratio, are inversely proportional to fecal tryptophan. The administration of prebiotics and probiotics, and fecal microbiota transplantation, could be possible effective therapies used for efficient management of obesity [36,61].

The location and function of EECs may be changed in obese people, which can result in dysregulated hormone release and difficulties controlling eating. Weight gain can result from EEC dysfunction, which can increase hunger and food intake.

Increased serotonin levels brought on by changes in the circadian rhythm may cause weight gain by causing symptoms of depression to manifest. So, national programs of psychotherapy for motivational counseling have led to improvements in weight loss, physical activity, and sleep quality, and the persistence of emotional eating tendencies should be under control in this way. These highlight the need for targeted strategies to address this behavior, in association with drug therapy.

Additionally, genetically targeted treatments for obesity are probably the most expected treatments for this illness, which is a hopeless situation for the majority patients.

In summary, future research regarding 5-HT’s involvement in obesity should include (1) larger studies to investigate the role of 5-HT in metabolic dysregulation; (2) studies of the influence of gut microbiota on TRP metabolism and the involvement of the IDO-1 pathway in metabolic alterations; (3) studies of the inflammatory diet’s effect on the cognitive impairment of eating disorders, along with 5-HT metabolism in this population; (4) development of more specific SERT evaluation in obese individuals; and (5) studies of the involvement of psychological distress in gut microbiota changes and 5-HT levels. Understanding all these pathways could represent therapeutic target areas in obesity management.

## 4. Conclusions

There is a complex and multidimensional relationship between obesity, serotonin levels, and weight regulation.

Regarding the correlations between the hypothalamus, leptin concentration, involved hormones, neurotransmitter reciprocations (especially that of serotonin), gut microbiota, and genetic predisposition, future targeted therapy should cover this complex axis, for an equilibrated balance in normal weight recovery or preservation, in the event of predisposing factors without an apparent clinical impact. For this purpose, ample and extensive studies are necessary to understand the complete mechanisms with the purpose of creating cost-effective molecular drug therapies for complete, reproducible treatment results.

## Figures and Tables

**Table 1:** Newcastle-Ottawa scale analysis of the included articles.

Author (Reference)	Selection				Comparability	Outcome			Total Score	Quality
	Representativeness of the Exposed Cohort	Selection of the Non-Exposed Cohort	Ascertainment of Exposure	Demonstration that Outcome of Interest Was Not Present at Start of Study	Comparability of Cohorts Based on the Design or Analysis	Assessment of Outcome	Was Follow-Up Long Enough for Outcomes to Occur	Adequacy of Follow-Up of Cohorts		
Huang et al. [29], 2024	*	*	*	*	*	*	*	*	8	Good
Kim et al. [30], 2023	*	*	*	*	*	*	*	*	8	Good
Liikonen et al. [31], 2024	*	*	*	*	*	*	*	*	8	Good
Borroni et.al [32], 2023	*	*	*	*	*	*	*	*	7	Good
Tkachenko et al. [33], 2023	*	-	*	*	*	*	*	*	7	Good
Tkachenko et al. [34],2023	*	-	*	*	*	*	*	-	6	Good
Castell et al. [35], 2022	*	-	*	*	*	*	*	*	8	Good
Dong et al. [36], 2022	*	*	*	*	*	*	*	-	7	Good
Orozco-Ruiz et al. [37], 2022	*	*	*	*	*	*	*	*	8	Good
Wu et al. [38], 2017	*	-	*	*	*	*	*	-	6	Good
Baumard et al. [39], 2021	*	*	*	*	*	*	*	*	8	Good
Van Galen et al. [40], 2021	-	-	*	*	*	*	*	*	6	Good
Hartstra et al. [41], 2020	*	*	*	*	*	*	*	*	8	Good
Palmeira et al. [42], 2019	*	*	*	*	-	*	*	*	7	Good
Fakhry et al. [43], 2018	-	*	*	*	-	*	*	-	6	Good
Nam et al. [44], 2018	*	*	*	*	*	*	*	*	8	Good
Groer et al [45], 2018	*	*	*	*	*	*	*	*	8	Good
Drabe et al. [46], 2017	*	*	*	*	*	*	*	*	7	Good
Ioannou et al. [47], 2016	*	-	*	*	*	*	*	*	7	Good

**Legend:** “*” indicates that the article meets the criteria mentioned above; “-” indicates that the article does not meet the above mentioned criteria.

**Table 2:** RoB2—risk of bias for RCT.

Article’s Title	Evaluation	Randomization Process	Deviations from Intended Interventions	Missing Outcome Data	Measurement of the Outcome	Selection of the Reported Results	Overall
Frigerio et al. [48], 2021		+	+	+	+	+	+
Grundmann et al. [49], 2020		+	+	+	+	+	+
Versteeg et al. [50], 2017		+	-	+	-	+	-

**Legend:** “+” indicates that the article meets the criteria mentioned above; “-” indicates that the article does not meet the above mentioned criteria.

**Table 3:** Characteristics of included studies in the systematic review.

Author, Year [Reference]	Participants * Study Design * Aim of the Study	Sample		Parameters	Outcomes	Discussions	Limitations	Future Directions
		Inclusion Criteria	Exclusion Criteria					
Huang, 2024 [29]	579 patients. * Cross-sectional study. * To investigate the correlation between DII and cognitive function.	45–75 yrs. Residents of 3 villages from Beijing. BMI = 18–24 kg/m^2^ or ≥28 kg/m^2^.	BMI <18 kg/m^2^ or 24–28 kg/m^2^. Severe thyroid, renal dysfunction. History of CNS or mental disease. Reading, hearing, visual impairment. Missing or mandatory suspicious data.	Anthropometric measurements. Cognitive function assessment (MoCA scale). Dietary inflammatory index (FFQ, DII). Metabolic biomarkers (FBG, lipid profile, ApoE, 5-HT). Inflammatory cytokines. Erythrocyte membrane fatty acid.	The 5-HT serum level was lower in patients with obesity compared to those with normal weight. Regarding diet type (anti-inflammatory, neutral and pro-inflammatory), there was no significant difference in 5-HT serum levels among the studied groups.	This study demonstrated a negative relationship between DII score and the cognitive function when comparing normal-weight to obese patients. The results suggest that erythrocyte membrane fatty acids could be the mediator for this result.	Small sample. Cross-sectional study. FFQ may result in recall bias. The result cannot be generalized because the study was conducted in a specific population.	Longitudinal and interventional studies are required to evaluate the association between DII and cognitive function, along with the cofactors implicated in this relationship.
Kim, 2024 [30]	290 patients. * Cross-sectional study. * To investigate the BMI and brain connectivity, exploring the neurotransmitter involvement and gene expression.	Healthy individuals from Human Connectome Project.	Genetically related Family history of mental illness History of drug abuse	MRI	Patients with increased BMI have a better navigation efficiency in the sensory, reward and executive control-related brain regions. All the modifications in navigation efficiency might be related to the serotonergic, dopaminergic, opioid and norepinephrine systems.	Patients with increased BMI had an altered brain communication pattern, particularly in the reward, cognitive and sensory-related regions. These relationships could be linked to dopaminergic, serotoninergic and cannabinoid systems.	Cross-sectional study. The participants were not followed-up. Did not account for others cofactors, such as eating disorders, or other communication models.	Longitudinal studies are necessary to evaluate the connection between BMI and brain connectivity changes over time, along with investigating supplementary network communications models, exploring brain networks associated with eating disorders, and potentially integrating multimodal imaging with molecular data.
Liikonen, 2024 [31]	79 patients. * Secondary analysis of a randomized controlled trial. * To investigate the correlation between whole-grain consumption, TRP metabolism, and psychological distress in a weight management intervention.	30–65 yrs. BMI 30–40kg/m^2^	BMI out of the inclusion range. DM Pregnancy. Kidney or thyroid dysfunction. Heart or liver diseases POS Diagnosed ED. Alcohol consumption. Neuroleptic medication. Oral cortisone medication.	GHQ Anthropometric measurements TRP metabolites	There was no significant association between whole-grain consumption and psychological distress. 5-HT levels increased after weight loss in patients with low psychological distress, as well as in those with high psychological distress, during the weight maintenance period. There were small differences over time in the serum levels of other TRP metabolites, but these differences were not statistically significant.	Whole-grain consumption influences TRP metabolism, potentially leading to positive outcomes for mental status. Psychological distress did not interfere with whole-grain consumption. TRP metabolism is directly influenced by weight loss and dietary changes, regardless of psychological discomfort.	Small sample The fiber content, not the percentage of whole grains in cereal items, served as the foundation for the study’s definition of whole grains. Self-reported food diaries which may contain misreporting were used to gauge consumption of whole grains.	Larger, long-term studies re necessary to assess the causality between whole grains, TRP metabolism, and psychological distress. It is important to evaluate all the cofactors involved, such as gut microbiota composition, gut-brain axis mechanisms, and the biochemical pathways linking weight-loss interventions to mental health.
Borroni, 2023 [32]	91 patients. * Cross-sectional study. * To assess the impact of night shift work on serum metabolites in a group of female nurses working in a hospital.	30–45 yrs. Nurse, with ≥1 year in service. Caucasian.	Male nurses. Insufficient metabolomics data.	BMI. Metabolomic profile (21 amino acids, 21 biogenic amines, hexoses, 40 acylcarnitine, 15 sphingolipids, and 90 glycerophospholipids). Years of night shift work experience. Different night shift schedules.	Low levels of glycerophospholipids and sphingolipids were detected in night-shift patients. Increased levels of taurine, 5-HT, aspartic acids were observed in night-shift patients.	This study compared the metabolic profile of individuals depending on the work schedule. The association between 5-HT, taurine, aspartic acid and schedule type was not statistically significant in this population. However, the differences between active night shift nurses compared with non-night shift nurses emphasize the potential disturbances associated with night shift work.	Small sample. Cross-sectional design. Population was composed only from women. Could not assess if the results are due to night shift work or the changes in behavioral. Could not be assessed if the metabolic reversible or if the exposure to night shift work affects metabolism over time. Unclear distinction between circadian disruption vs behavioral changes in metabolism changes. The blood sample collection (for night shift workers – at the end of the shift, for non-shift workers – at the start of the workday).	Larger studies, including male participants, are needed. Future research should also evaluate differences between various shift schedules and determine whether observed metabolic changes result from direct circadian rhythm disruption or altered behaviors.
Tkachenko, 2023 [33]	75 patients. * Prospective, cohort study. * To determine the correlation between body weight, 5-HT levels, mental health, sleep disturbances and metabolism in patients with obesity.	BMI = 25–39.9 kg/m^2^. 25–54 yrs.	BMI <25 kg/m^2^ or >40 kg/m^2^. <25 yrs. >54 yrs. Pregnancy or breastfeeding. History of allergic reactions. Chronic diseases (POS, hypothyroidism, Cushing’s syndrome, DM, resistant HTN, CKD stage 2–5).	Anthropometric measurements. BP. Psychological and sleep assessment (HADS, HAM-A, ESS, PSQI). QoL-SF-36 questionnaire. Metabolic panel (lipid profile, FBG, insulin, leptin, 5-HT).	Strong correlations were observed between BMI and anthropometric measurements, BP, lipid profile, and psychological assessment scores (HADS, BDI, HAM-A, and ESS). At baseline, 5-HT levels were inversely associated with all studied parameters. After 6 months of therapeutic intervention, 5-HT levels significantly increased in the obese group (*p* < 0.001) but showed no significant change in normal-weight patients (*p* > 0.05).	Sleep disorders contribute to the obesity’s development. Decreased 5-HT levels interfere with mood disorders, lipid and carbohydrate metabolism and contribute to the increased BMI.	No control group. Short follow-up period (6 months). Results are part of a larger ongoing study, published separately. Self-reported sleep and mental health data may introduce bias. Results cannot be generalized to overweight or normal-weight individuals, as the study population consisted exclusively of obese patients.	Longer follow-up studies and larger sample sizes are needed to better understand the metabolic and psychological effects of obesity. Additionally, further investigation into 5-HT’s role in weight management and the mechanisms of obesity is required.
Tkachenko, 2023 [34]	37 patients. * Prospective observational study. * To evaluate the impact of patient-centered motivational therapy on lifestyle improvements in obese individuals.	BMI = 30–40 kg/m^2^. Prime working age.	Severe comorbidities (DM, cardiovascular, thyroid disorders). Pregnant or breastfeeding.	Anthropometric measurements. Clinical examination. Metabolic parameters (FBG, insulin, HOMA index, lipid profile, leptin, 5-HT). Cognitive and depression questionnaires.	At the first visit, serotonin levels were low. At the 1- and 3-month visits, serotonin levels increased, but the change was not statistically significant (*p* > 0.05). After 6 months, 5-HT levels increased significantly compared to the first visit (*p* < 0.05). Other parameters, including BMI, BP, glucose, HDL, VLDL, as well as cognitive and depression questionnaire scores, showed significant improvement (*p* < 0.05).	The patient-centered approach improves 5-HT levels, along with most of the other parameters assessed in this study, suggesting potential neuroendocrine benefits	Small sample. No control group. Short period of follow-up. Self-reported data (such as mental health, eating behavior, physical activity) increase the risk of bias.	Longer follow-up studies and larger sample sizes are needed to better understand the metabolic and psychological effects of obesity. Additionally, further investigation into 5-HT’s role in weight management and the mechanisms of obesity is required.
Castell, 2022 [35]	13 children (8–15 yrs.). * Experimental study. * To investigate the β-cell mass expansion in puberty using human pancreatic samples and rat models.	Postmortem pancreatic samples form 13 children. Wistar rats used for β-cell proliferation during puberty * Wistar rats at weaning, puberty, and young adulthood. Groups fed with normal or high-fat diet.	Adults or children outside the 8–15-year range. Patients with DM or pancreatic disease. * Rats with developmental abnormalities.	β-cell proliferation. Metabolic markers (glucose, insulin sensitivity, GH, IGF-1, 5-HT). Gene expression (GH/GHR/5-HT signaling pathways).	β-cell proliferation was stimulated by puberty in both rats and humans. During puberty, increased GH levels stimulate 5-HT production in pancreatic β-cells. β-cell proliferation was inhibited by blocking HTR2B signaling, suggesting that 5-HT acts through the HTR2B receptor. A high fat diet suppressed β-cell proliferation, resulting in metabolic disturbances, such as glucose intolerance and impaired insulin secretion.	β-cell proliferation during puberty is stimulated through GH and 5-HT signaling. Impairment of β-cell proliferation, due to obesity and high-fat diet, leads to glucose intolerance. Any changes in pubertal period increase the risk of metabolic disorders in adulthood, especially T2DM.	Small size for human pancreatic samples. Cross sectional human study. High-fat diet experiments were limited to male rats. Did not directly measure insulin secretion in humans, only β-cell markers.	Longitudinal studies are needed to track β-cell changes over time in humans. Furthermore, interventional studies should assess whether the GH/5-HT signaling pathways, along with pharmacological modulation of HTR2B, can influence β-cell proliferation and reduce the risk of DM.
Dong, 2022 [36]	287 patients. * Cross-sectional study. * To identify association between obesity and BGM signature, evaluating the gut microbiota composition, brain network activity and metabolites.	Right-handed patients. No medical or psychiatric diseases.	Pregnant or breastfeeding. Substance or tobacco use. Chronic disorders (neurological, psychiatric, metabolic disorders). Extreme workout (>8 h/week). Chronic medication (that interfere with CNS, analgesic drugs). No administration of antibiotic 3 months prior to admission.	MRI. Anthropometric measurements. Stool sample (16s ribosomal RNA gene sequencing, metabolite analysis). Diet surveys.	Altered brain connectivity, especially in the reward system, observed in patients with obesity, suggests appetite dysregulations. Fecal levels of TRP were decreased in obesity group. The *Prevotella/Bacteroides* (P/B) ratio was elevated in obesity group. The correlation between P/B ratio and nucleus accumbens centrality was positive (*p* = 0.03). The correlation between P/B ratio and fecal TRP levels was negative (*p* = 0.004).	In individuals with obesity, alterations of brain network activity, particularly in appetite regulation centers, imply a distinct BGM signature, regarding of race, sex, and diet. An increased P/B ratio, along with increased nucleus accumbens activity, are obesity risk factors.	Cross sectional study design. Self-reported dietary data may induce bias. Results may be influenced by racial differences. 16s rRNA sequencing has limited resolution in terms of species- and strain-level analyses. Metabolic hormones were not assessed. No longitudinal follow-up to evaluate the long-term effects of microbiome-brain interactions.	Longitudinal studies are needed to assess the relationship between the microbiome and brain activity. Moreover, gut microbiome composition should be analyzed to explore potential mechanisms for weight management strategies.
Orozco-Ruiz, 2022 [37]	1790 patients. * Cross-sectional study. * To investigate whether VAT and SAT influence metabolic health through specific metabolite concentrations, including BCAA and AAA. Additionally, the study explored the effects of VAT on insulin resistance, inflammation, and metabolic risk.	>30 yrs. Participants from the Rhineland Study with available MRI and blood metabolomics data.	Participants with extreme values in metabolite concentration and cardiometabolic risk markers. Participants without valid data on abdominal MRI-fat segmentation.	Anthropometric measurements. Assessment of physical activity, cardiovascular health, cognitive, neurological, ophthalmological function. Metabolomics analysis (TRP, 5-HT, tyrosine, methionine, KYN, 5-hydroxyindole-acetic acid, DA) Cardiometabolic risk markers (insulin, lipid profile, FBG, hs-CRP, BP). MRI.	BCAA and AAA increased levels were observed in patients with metabolic risk factors. VAT correlated with systemic inflammation through KYN, as the primary metabolite mediating. VAT volume correlated with high levels of AAA (L-isoleucine, L-leucine, indole-3-lactic acid). Indole-3-propionic acid was inversely correlated with VAT. TRP metabolites were correlated with inflammation biomarkers. 5-HT was associated with an increased risk of hypertriglyceridemia in men compared to women.	BCAA and AAA exhibit increased serum levels in patients with elevated VAT. In this group, both BCAA and AAA, mediate VAT’s implication on metabolic pathways. 5-HT metabolism shows sex-specific effects on lipid metabolism.	Cross-sectional study design. Lack of tissue-specific metabolomics; adipose tissue metabolic activity was not directly assessed. Self-reported dietary data may induce bias. The study did not account for genetic factors or gut microbiota contributions to metabolite variation. 5-HT metabolism exhibits sex-specific effects on lipid metabolism, requiring further investigation.	Longitudinal studies are needed to assess the causal effects between VAT and metabolite alterations over time. These studies should integrate genetic and microbiome analyses to better characterize their role in obesity development. Further research is required to investigate sex differences in 5-HT metabolism and lipid regulation.
Wu, 2017 [38]	20 patients. * Cross-sectional study. * To assess the SERT in patients with morbid obesity and ED using SPECT imaging.	Young adults. Morbid obesity. No history of ED.	History of psychiatric or neurological disorder. Head trauma. Cardiometabolic disorders (HTN, DM). Anorexic medications use. Use of any systemic medication in the last 4 weeks. Smoking or alcohol abuse. Participation in a weight-loss trial in the past 12 months.	SCOFF questionnaire. SPECT. DXA. Anthropometric measurements.	No significant correlations between SERT and age, BMI and BF distribution.	No significant correlations between midbrain SERT availability and BMI or BF. No significant differences were observed in radiotracer delivery (10-min MID/CE ratio) and SERT availability (6-h MID/CE ratio) between morbidly obese and non-obese individuals without ED.	Small sample. Cross-sectional study design. Not included patients with BMI between 25–39.9 kg/m^2^. Did not measure 5-HT levels. SPECT is not the best imagistic method to assess the serotoninergic regulation. Others regulatory systems, such as dopaminergic, noradrenergic or leptin levels were not evaluated.	Larger longitudinal studies with a broader BMI range are needed to track SERT changes in response to weight fluctuations. Also, these studies should evaluate dietary factors, gut microbiota, and hormonal influences. Furthermore, assessing 5-HT receptor function instead of SERT may provide a more accurate tool for understanding these correlations.
Baumard, 2021 [39]	67 patients. * Cross-sectional study. * To determine whether obesity affects the expression of EECs and nutrient-sensing G-protein-coupled receptors in the human colon. Additionally, it assessed whether alterations in gut-derived satiety hormones contribute to appetite dysregulation in obesity.	Adult patients undergoing colorectal surgery or colonoscopy.	Patients with inflammatory bowel disease, active cancer, intestinal inflammation. Patients using medications that affect gut hormone levels.	Gene expression analysis (mRNA levels of nutrient receptors, mRNA levels of satiety hormones). Protein expression. BMI and colonic tissue sampling.	No significant differences in gut hormone expression between obese and non-obese participants regarding mRNA levels of 5-HT, glucagon, PYY, cholecystokinin, and somatostatin were unchanged between BMI groups. Also, no significant changes in enteroendocrine cell numbers between BMI groups. Obesity was associated with increased GPR40 expression in the sigmoid colon. No significant difference in 5-HT -positive EECs or PYY-expressing L-cells in the colon, but calcium-sensing receptor was highly expressed on 5-HT -positive EECs, regardless of BMI.	In this study, obesity does not significantly influence EECs numbers or satiety hormone expression in the human colon. The results suggest that appetite dysregulation present in obesity group is not caused by alteration of gut hormone production, but may involve impaired hormone release or receptor sensitivity.	Small sample size. Cross-sectional study design. Assessment was limited to mRNA and protein expression; functional hormone secretion was not measured. Differences in patient group. No followed-up of patients. Hormone release from EECs and their activation in response to nutrient binding were not evaluated.	Others studies are necessary to establish the influence of gut microbiota and dietary components on nutrient-sensing receptors. Moreover, it is mandatory to assess the impact of obesity management interventions, such as weight loss and bariatric surgery, on gut microbiota components and hormone function.
Van Galen [40]	20 subjects. * Randomized controlled crossover study. * To investigate the effects of 12-hour vs. 24-hour fasting on SERT and DAT availability in the hypothalamus/ thalamus and striatum using SPECT imaging, comparing lean vs. obese individuals.	Men. 50–75 yrs. BMI <25 kg/m^2^ and >30 kg/m^2^. Stable weight (<5% weight change in the last 3 months).	Use of any medication (except for thyroid hormone, antihypertensive, and/or lipid-lowering drugs). History of any psychiatric or eating disorder. Shift work, irregular sleeping habits, regular vigorous exercise. Substance abuse (smoking, alcohol >3 units/ day, and/or recreational drugs). Any contra-indication for MRI.	SPECT. Metabolic markers (FBG, insulin, glucagon, leptin, ghrelin). FFA.	Patients with normal BMI showed a significant increase in hypothalamic SERT availability after 24-hour fasting (*p* = 0.044), with no significant changes in the obesity group. Lean men had a significant drop in insulin and leptin compared to obese individuals. Increased FFA serum levels positively correlated with hypothalamic SERT availability and negatively correlated with striatal DAT availability. DAT availability did not significantly differ between fasting conditions.	Regarding of fasting effect, a 24-hour fast increases hypothalamic SERT availability in patients with normal BMI, with no effect in patients with obesity. The results from this study suggest that metabolic dysregulations observed in patients with obesity may influence the serotoninergic system, being a potential mechanism in obesity development.	Small sample size. All participants were male, so findings cannot be generalized to females. Participants had mostly Class I obesity, meaning results may not apply to severe obesity. Did not measure actual 5-HT or dopamine levels, only transporter availability. SPECT imaging resolution limits detailed analysis of neurotransmitter subregions.	Larger studies should investigate whether fasting may induce changes in serotonin signalling, and if these changes could predict weight loss outcomes. Also, these studies should include both male and female, to proper evaluate the sex-specific differences. Further exploration of 5-HT and DA receptor function in obesity is necessary.
Hartstra et al., 2020 [41]	24 patients. * Double-blind randomized controlled intervention trial. * To investigate the effects of FMT from post-RYGB donors versus oral butyrate supplementation on the gut-brain axis, DAT and SERT binding, and metabolic outcomes in individuals with MetS.	50–70 yrs. MetS.	Use of any medication in the last 3 months. Pre- and/or probiotics use. Substance abuse. eGFR <60 mL/min/1.73 m^2^. Contraindications for MRI. History of cardiovascular event. History of psychiatric disorder.	SPECT. Metabolic markers (FBG, insulin, HOMA-IR, plasma metabolites, resting energy expenditure and intestinal transit time, urinary 5-HIAA). Gut microbiota analysis (16S rRNA sequencing).	FMT from post-RYGB donors increased striatal DAT binding, while butyrate supplementation reduced DAT binding (*p* = 0.02). SERT binding in the hypothalamus showed a positive trend after FMT, but this was not significant. No significant effects of FMT or butyrate on weight or insulin sensitivity. Increased *Bacteroides uniformis* was associated with higher DAT binding, while increased *Prevotella* spp. was linked to lower DAT binding. Glycine, betaine, methionine, and lysine levels (metabolites involved in 5-HT and DA synthesis) were associated with changes in DAT expression.	Changes in gut microbiome were associated with alteration of DAT binding, with no effect on SERT availability. FMT increased brain DAT binding, while butyrate supplementation reduced DAT binding Despite these results, neither FMT or butyrate administration significantly influenced insulin sensitivity and body weight.	Small sample size. Cross-sectional study. No follow-up. No normal-weight control group. Did not measure actual neuro-transmitter levels, only transporter binding. Limited dietary control beyond monitoring via self-reported food diaries.	Larger, long-term studies are needed to confirm the gut-brain effects of FMT and butyrate. Including a normal-weight control group would help clarify obesity-related differences. Investigating whether modifying gut microbiota could serve as a therapeutic strategy for metabolic and neuropsychiatric disorders is essential.
Palmeira, 2019 [42]	93 patients. * Case-control study. * To investigate the association of genetic variants associated with BED in overweight/obese women.	20–58 yrs. BMI >25 kg/m^2^. Diagnosed BED.	Men. Other psychiatric condition than BED. Use of weight-loss medication or prior bariatric surgery.	Genetic polymorphisms (FTO, SLC6A4, DRD2, BDNF, GHRL). Psychological and behavioral assessment (EDE, BES). Anthropometric measurement. Metabolic biomarkers (FGB, lipid profile).	No significant association between any of the genetic variants and BED. Lower frequency of the FTO obesity risk in BED patients than in controls. SERT gene showed no association with BED (*p* = 0.689). DRD2 gene also showed no association with BED (*p* = 0.278). BDNF polymorphisms did not significantly differ between groups. GHRL polymorphisms were not associated with BED.	No correlations were found between BED and genetic polymorphisms (FTO, SLC6A4, DRD2, BDNF, or GHRL). The FTO gene, linked to obesity risk in other studies, was less frequent in BED patients than in controls.	Small sample size. No men included. No functional analysis of genetic polymorphisms’ implication on gene expression or metabolism. Findings contrast with previous studies linking FTO rs9939609 to other eating disorders (AN and BN). No interactions’ assessment between genetics and environmental factors (e.g., diet, stress).	Longitudinal research is necessary to determine the effect of genetic and environmental factors in the development of BED. Also, evaluating the impact of genetic variants on brain function and appetite regulation could provide new information regarding BED mechanisms and possible therapeutical approaches.
Fakhry, 2018 [43]	6 patients. * Observational, descriptive study. * The characterization of different types of ECs in the human gastric fundus and corpus and to examine the relationships between EECs and other gastric mucosal cells.	48–60 yrs. Gastric resection (greater curvature). Non-diabetic patients.	Diabetes. Patients with inflammatory or cancerous gastric conditions. History of prior gastric surgery.	Immunohistochemistry for specific ECs markers (ghrelin, 5-HT, somatostatin, PYY, GLP-1, gastrin and pancreastatin).	In four cases, through IHC methods, were found ghrelin, pancreastatin-, 5-HT-, and somatostatin-immunoreactive cells. In the process of calbindin, PYY and GLP-1 detections, few cells presented the last two searched markers. It was not obtained the IMR for calbindin in analyzed probes. It was used anti-H/K ATPase antibody to mark the parietal cells. 5-HT cells and somatostatin cells were less dense, compared to both ghrelin and pancreastatin in each region. 5-HT cells were evidenced by anit-5-HT antibodies – without IMR in mast cells, and positive IMR in EECs. 5-HT and somatostatin presented a small degree of colocalisation.	5-HT and somatostatin cells are often closely associated with parietal cells, suggesting possible paracrine regulation of acid secretion. Enteroendocrine-like cells contain both pancreastatin and 5-HT, suggesting a novel subpopulation of 5-HT-releasing gastric cells.	Small sample size. Cross-sectional study design. No followed-up. Only obese patients undergoing surgery were included, excluding non-obese or diabetic individuals. No functional analysis of hormone secretion—only cell morphology and distribution were examined. Did not analyze how EECs distribution varies with diet, metabolic status, or obesity severity.	New research is needed to evaluate the role of gastric EECs in appetite regulation, acid secretion, and digestion. Research should investigate EECs’ changes in metabolic disorders such as DM, obesity, and after bariatric surgery. Further exploration of gut-brain signalling pathways involving 5-HT and ghrelin is essential.
Nam, 2018 [44]	192 patients. * Cross-sectional study. * To evaluate the relationship between DAT and SERT availability in the brain and obesity.	>30 yrs. No history of neurological disorders. No use of medication affecting DAT or SERT.	Neurological disease. First-degree relatives with Parkinson’s disease. MoCA ≤ 26. Anticoagulation. Use of investigational drugs.	SPECT. Anthropometrics measurements.	Negative correlation between BMI and midbrain SERT (*p* = 0.0496) and positive correlation between BMI and midbrain SERT in non-obese individuals (*p* = 0.0053). Higher BMI was associated with increased SERT binding in the pons (*p* = 0.0026). DAT availability was not significantly correlated with BMI. SERT availability was significantly higher in men than women (*p* < 0.05). DAT availability decreased with age in the caudate nucleus (*p* = 0.0001), striatum (*p* = 0.0022) and thalamus (*p* = 0.0074).	Obesity influences midbrain SERT availability. Striatal DAT availability showed no correlation with BMI, whereas pontine SERT availability was positively correlated with BMI. SERT availability was higher in men, while DAT availability showed no sex-related differences. Both DAT and SERT exhibited age-related declines, consistent with previous studies.	Cross-sectional study design. No longitudinal follow-up to assess whether changes in neurotransmitter transporters predict weight gain or loss. Did not assess behavioral measures (e.g., appetite control, food reward sensitivity). BMI was used as the primary obesity measure, but fat distribution (visceral vs. subcutaneous fat) was not examined. Only transporter availability was assessed.	Further studies are needed to explore the associations between neuroimaging findings and BMI, specifically examining the effects of food intake, food stimulation, and glucose loading in both obese and lean individuals. Longitudinal studies are needed to determine whether SERT changes contribute to obesity development or result from obesity. Future research should incorporate additional measures, such as gut microbiota analysis and dietary intake, to better assess gut-brain interactions. Investigation into how 5-HT receptor function (5-HT1B, 5-HT2C) differs between obese and non-obese individuals is essential.
Groer, 2018 [45]	374 participants. * Cross-sectional study. To investigate whether obesity-induced inflammation influences the TRP-KYN pathway and its potential effects on 5-HT availability, depression, and metabolic function in pregnancy.	Pregnant women. 2nd trimester. Prepregnancy BMI availability to classify participants into groups.	Drug or alcohol abuse. Autoimmune disease. Previous thyroid disease. Use of medication that could affect the immune function. BMI <18 kg/m^2^. In vitro fertilization, multiple pregnancies	Anthropometrics measurements. TRP metabolism (TRP, KYN, KYN/TRP ratio as an index for IDO-1 enzyme activation). Profile of mood state depression/dejection subscale instrument (POMS-D). Inflammatory biomarkers (neopterin, nitrite levels).	Patients with obesity before pregnancy experienced an increased weight during pregnancy compared with overweight. Women with obesity had significantly lower TRP levels compared to non-obese women (*p* < 0.01). KYN/TRP ratio was significantly higher in obese vs non-obese patients (*p* < 0.01) Nitrite levels was higher in patients with obesity (*p* < 0.05). TRP levels was lower in patients with high POMS-D score (*p* = 0.017).	In this study, KYN/TRP ratio was used as a substitute for IDO-1 activity. Increased IDO-1 activity may reduce 5-HT availability, contributing to mood disturbances and systemic inflammation. The findings support the hypothesis that systemic chronic inflammation from obesity influences 5-HT synthesis, which might increase the risk of depression and metabolic dysfunction during pregnancy.	Cross-sectional study design. The participants were not followed during pregnancy and postpartum. Lack of inflammatory markers measurements. Did not directly measure 5-HT or its metabolites. Did not assess dietary intake, which could influence TRP and KYN levels. POMS-D was not validated in pregnancy.	Longitudinal studies are needed to track TRP metabolism throughout pregnancy and postpartum. Moreover, measuring 5-HT and its metabolites is essential to confirm the effects of TRP depletion or IDO-1 pathway of TRP’s metabolization. Further research should investigate the influence of diet and the microbiome on TRP metabolism.
Drabe, 2017 [46]	44 patients. * Cross-sectional study. * To investigate DNA methylation of the SERT gene and its relation to SERT availability and reward function in obesity.	21–59 yrs. BMI >35 kg/m^2^. No history of psychiatric disorder or major metabolic disease	Alcohol or drug abuse. Neurological or psychiatric disorders. Current use of medication affecting brain 5-HT function. Diabetes (insulin-dependent or poorly controlled). Contraindications for MRI.	PET. Genetic and epigenetic assessments (SERTLPR genotyping, DNA methylation). Reward sensitivity assessment (BAS, BIS). Anthropometric measurements. Metabolic biomarkers (FBG, insulin, HbA1c).	Increased levels of SERTLPR were associated with lower SERT availability in the prefrontal cortex of obese individuals (*p* < 0.05). Higher rates of CpG10 methylation in the SERTLPR gene were associated with greater reward sensitivity in obesity (*p* = 0.001). SERTLPR methylation was not directly correlated with BMI values.	The results from this study highlights the obesity implication in SERT availability, by epigenetic modifications of the SERT gene, along with neurobiological alteration in the reward sensitivity center. These findings explain the compulsive eating in patients with obesity.	Small sample size. Cross-sectional study design. Peripheral blood leukocytes were used to assess SERTLPR methylation, which may not fully reflect brain methylation patterns. The exclusion of SERTLPR/rs25531 genotype. Did not investigate the 5-HT receptor system or 5-HT metabolism. No dietary intake or eating behavior assessment beyond BAS/BIS scales.	Longitudinal studies are needed to assess whether SERTLPR methylation changes over time with weight gain or loss. Research should explore the impact of dietary and behavioural interventions on 5-HT -related reward processing. Further investigation is required to determine whether pharmacological or lifestyle interventions targeting 5-HT signaling can reduce ED in obesity.
Ioannou, 2016. [47]	14 patients. * Randomized, placebo-controlled study. * To determine whether 5-HTP influences brain activity in response to food stimuli, potentially affecting food preferences.	Patients in the mid-20s of life. No physical, neurological, or ED. Not taking any medications affecting 5-HT function.	Psychiatric or neurological disorders. History of ED. Chronic medication.	Functional MRI. Food stimuli categories (visual stimuli). Macronutrient preference and food recall.	In the 5-HT group, patients exhibited increased activation in prefrontal cortex, limbic structures and basal ganglia, compared to Vitamin C group. In the Vitamin C group, increased activation was observed in visual processing areas, including the fusiform gyrus, occipital lobe, and temporal gyrus. Not statistically significant difference between caloric intake recalls between 5-HT group and Vitamin C group.	The results of this study sustain the brain activation signature differs according to food preferences, along with 5-HT role in regulating food selection and eating behaviors. Patients from 5-HT group exhibited a stronger preference for protein-rich foods, and patients from Vitamin C group, for high-calorie and carbohydrate-rich food.	Small sample size. Short-term study. Did not measure actual food intake after scanning, only food recall and brain activity. Did not directly measure 5-HT levels in blood or cerebrospinal fluid. Limited control over dietary habits before the experiment.	Long-term studies are needed to assess the sustained effects of 5-HT on food preferences and weight management. Moreover, direct measurement of 5-HT levels is necessary to establish its implication and possible mechanism.
Frigerio, 2021 [48]	1391 patients. * Cross-sectional study. * To identify the metabolome profile of individuals with overweight and obesity and to explore the association between metabolites and obesity-related metabolic dysfunction.	>18 yrs. BMI > 25 kg/m^2^. Residents of Lombardy, Italy. [51]	Pregnant or breastfeeding women. History of cancer, heart disease, stroke, multiple sclerosis, Alzheimer’s disease, Parkinson’s disease, psychiatric diseases, epilepsy.	Anthropometric measurements. Metabolomic profile (amino acids, biogenic amines, acylcarnitine, sphingolipids and glycerophospholipids, hexose)	In this study, patients had increased BCAA, AAA, KYN, and hexoses serum levels, along with decreased phosphatidylcholines, lysophosphatidylcholines, and 5-HT serum levels. Increased levels of BCAAs and AAAs were positively correlated with metabolic risk factors (BMI, insulin resistance). Lipid profile modification correlated with higher BMI. 5-HT levels were negatively associated with BMI. KYN was positively associated with BMI.	The results of this study highlighted that obesity is associated with increased serum levels of BCAA, AAA, and glucose-related metabolites. Also, low serum levels of 5-HT levels correlate with obesity, suggesting a possible pathway between neurotransmitter function and obesity.	Cross-sectional study design. Did not measure hormone levels (insulin, leptin, cortisol) to link metabolism with obesity. The metabolome is influenced by diet. The majority of patients were female. No correlation between metabolomic profile and metabolic status.	Longitudinal studies are needed to assess metabolic changes over time in obesity progression, along with the incorporation of dietary intake and microbiome analysis to refine metabolic associations. Additionally, investigating 5-HT’s role in both the metabolic and psychological aspects of obesity may be crucial in understanding obesity-related metabolic risk
Grundmann, 2020 [49]	37 patients. * Cross-sectional study. * To evaluate the association between central SERT and HbA1c levels in patients with obesity and without T2DM.	Adults with obesity (BMI > 35 kg/m^2^). HbA1c ≤ 5.9%.	Obesity due to prediabetic metabolism. History of psychiatric or neurological disease. Family history of psychiatric disease. Metabolic risk factors (resistant HTN, DM). Centrally-acting medication. Diet in the last 6 months. Alcohol or drugs use. Pregnancy or breastfeeding. Contraindication for MRI.	Blood biomarkers (HbA1c, BDNF). SERT. PET.	Increased HbA1c levels are associated with lower hippocampal SERT availability in obesity individuals. In patients with structural changes in the hippocampus were observed increased serum levels of HbA1c. In individuals with the S allele of the SERT gene, higher values of HbA1c correlated with lower BDNF levels. No significant differences in overall SERT availability between obese and non-obese individuals.	The results of this study suggest that chronic high blood glucose levels may be involved in hippocampus’ microstructural changes, and possible alteration of 5-HT signaling pathways. The effects of these alterations imply cognitive, appetite, and metabolic dysregulations. Also, BDNF could be the connection between glucose metabolism and hippocampal 5-HT function, especially in individuals with certain genetic variants.	Small sample size. Cross-sectional study design. Only non-diabetic individuals were included, so findings may not apply to obese individuals with diabetes. Did not measure FBG or insulin, which could further clarify metabolic dysfunction. No direct assessment of 5-HT levels, only SERT availability.	Longitudinal studies are needed to assess whether high levels of HbA1c interfere with 5-HT changes, and these could predict cognitive impairment or metabolic dysfunction over time. Including patients with T2DM is essential to establish whether these modifications are more important in diabetic populations. Further research should explore gut-brain interactions, particularly the microbiome’s influence on 5-HT signaling.
Versteeg et al. 2017 [50]	20 patients. * Case-control study. * To investigate whether SERT and DAT binding differ in lean, ISO, and IRO individuals.	21–59 yrs. BMI >35 kg/m^2^. Normal liver, renal, thyroid function. Non-smokers. No medication use. Stable weight in the last 3 months.	Pregnancy. History of psychiatric, eating disorders. Shift workers. Substance abuse.	SPECT. Metabolic biomarkers (FBG, insulin, C-peptide levels). Anthropometric measurements.	SERT binding in the diencephalon was significantly lower IRO individuals compared to ISO (*p* = 0.009) and lean individuals (*p* = 0.019). Not statistically significant difference between the three groups regarding hypothalamic SERT binding (*p* = 0.059). No difference between groups regarding DAT availability in the striatum. Lower diencephalic SERT binding correlated with increase HOMA-IR score.	The results of this study highlight a possible link between 5-HT signaling and insulin resistance, independent of body weight. Reduced 5-HT function in patients with increased values of HOMA-IR, regardless of their BMI, could explain metabolic modifications along with the 5-HT implication in insulin sensitivity.	Small sample size. The radiotracer is not selective regarding SERT vs DAT binding. Did not measure 5-HT levels directly—only transporter availability. Did not assess dietary intake, which could influence 5-HT function.	Longitudinal studies are needed to assess whether SERT availability changes in response to metabolic interventions. Also, advancements in imaging techniques are needed to improve the analysis of SERT availability.

**Legend:** * = element used for separating the main characteristics of the study (found in the second coloumne of the table); 5-HT = serotonin or 5-hydroxytryptamine;AAA = aromatic amino-acids; AN = anorexia nervosa; ApoE = apolipoprotein E; BAS = behavioral activation system;BCAA = branched-chain amino-acids; BDI = Beck’s Depression Inventory; BDNF = brain-derived neurotrophic factor; BED = binge eating disorders; BF = body fat; BGM = brain-gut microbiome; BIS = behavioral inhibition system; BMI = Body Mass Index; BN = bulimia nervosa; BP = blood pressure; CKD = chronic kidney disease; CNS = central nervous system; DA = dopamine;DAT = dopamine transporter; DII = dietary inflammatory index; DM = diabetes mellitus; DNA = deoxyribonucleic acid; DRD2 = dopamine D2 receptor; DXA = dual-energy X-ray absorptiometry; ECs = endocrine cells; ED = eating disorders; EECs = enteroendocrine cell; eGFR = estimated glomerular filtration rate; ESS = Epworth Sleepiness Scale; FBG = fasting blood glucose; FFA = free fatty acids; FFQ = food frequency questionnaire; FMT = fecal microbiota transplantation; FTO = fat mass and obesity-associated gene; GHQ = General Health Questionnaire-12; GHRL = ghrelin gene polymorphism; GLP-1 = Glucagon-like peptide 1; HADS = Hospital Anxiety and Depression Scale; HAM-A = The Hamilton Scale; HbA1c = hemoglobin A1c; HOMA-IR = Homeostatic Model Assessment for Insulin Resistance; HTN = arterial hypertension; IDO-1 = indoleamine 2,3-dioxygenase 1; IHC = immunohistochemistry; IMR = immunoreaction; IRO = insulin-resistant obese; ISO = insulin-sensitive obese; KYN = kynurenine; MetS = metabolic syndrome; MoCA = Montreal Cognitive Assessment; MRI = Magnetic Resonance Imaging; mRNA = messenger ribonucleic acid; PET = Positron Emission Tomography; POS = Polycystic ovary syndrome; PSQI = Pittsburgh Sleep Quality Index; PYY = YY Peptide; QoL-SF = quality of life SF questionnaire; rRNA = ribosomal RNA; RYGB = Roux-en-Y gastric bypass; SAT = subcutaneous adipose tissue; SERT = Serotonin Reuptake Transporter; SLC6A4 = solute carrier family 6 member 4 (SERT); SPECT = Single-Photon Emission Computed Tomography; T2DM = type 2 diabetes mellitus; TRP = tryptophan; VAT = visceral adipose tissue; yrs = years.

**Table 4:** A structured synthesis of elements found in the relevant selected articles.

Mechanism	Features	Reference
Metabolic factors	Increased BCAA concentrations can lead to Insulin resistance.	[37,48]
	Dysregulation of BCAAs pathways lead to serotonin signaling impairment.	[36,37,48]
	5-HT, GH and IGF-1 interfere with β-cell mass expansion leading to obesity and an increased risk of T2DM later in life.	[35]
	Decreased levels of 5-HT correlate with mood disturbances and overeating.	[33,34,35]
	Microbiome rich in *Prevotella* spp. contribute to insulin resistance via BCAA production.	[36]
	Insulin resistance is associated with impaired 5-HT signaling and chronic inflammation.	[45,50]
	Serotonin-dopamine dysregulation leads to increased appetite for high-calorie food, leading to obesity.	[30,33,34]
Microbiota and diet habits	5-HT levels are decreased in patients with obesity, being influenced by diet habits and sleep disturbances.	[29,32]
	Peripheral serotonin influences the brain-serotonin via vagal signaling.	[31,35,48]
	Tryptophan metabolism dysregulation decreases the brain serotonin production.	[31,36,37,45,48]
	High-fat diets contribute to microbiota dysregulation, which maintains low-grade inflammation through increased *Prevotella* spp.	[36]
	SCFAs improve serotonin signaling	[41]
Genetics	Genetic variants such as rs6265 (for BDNF) or SLC6A4 (for 5-HTTLPR), are genetic variations which affect serotonin signaling and may contribute to obesity through increased appetite.	[42,46]
Neurotransmitters	Dopamine and serotonin modulate motivation and reward mechanisms through 5HT receptors and D1 receptors.	[30,44,47]
	Serotonergic agonists via 5-HT1B and 5-HT2C receptors and antagonism via 5-HT6 receptor lead to an anorexic effect.	[38]
	Enterochromaffin cells release serotonin in response to gastrointestinal tract content.	[39,43]
	High levels of HbA1c interfere with SERT availability in the hippocampus of patients with obesity. BDNF is involved in pathogenesis of T2DM, depression and dementia.	[49]
	In obesity, fasting did not increase SERT availability, suggesting a chronic perturbation in serotonergic signaling.	[40]
	BMI influences brain communication pattern, through serotoninergic and dopaminergic systems.	[30]
	Serotonin and dopamine dysregulations contribute to eating disorders and obesity.	[46]

**Legend:** 5-HT, 5-hydroxytryptamine; 5-HT1B, 5-hydroxytryptamine receptor 1B; 5-HT2C, 5-hydroxytryptamine receptor 2C; 5-HTTLPR, serotonin transporter-linked promoter region; BCAA, branched-chain amino acid; BDNF, brain derived neurotrophic factor; GH, growth hormone; HbA1c, hemoglobin A1c; IGF-1, insulin-like growth factor-1; SERT, Serotonin Reuptake Transporter; SLC6A4, solute carrier family 6 member 4; T2DM, type 2 diabetes mellitus.
